# An Entering Wedge,—Perhaps

**Published:** 1900-09

**Authors:** 


An Entering Wedge,—Perhaps.—At the Democratic State Convention held at Waco August 8th to 10th, ult., active advocates of a State Board of Health and Vital Statistics succeeded in getting the platform committee to adopt a resolution favoring the passage of a bill by the next Legislature, creating such a board. Dr. Harrison, of Columbus, Texas, chairman of the State Medical Association's committee on State Board of Health, is entitled, in great part, to the credit for it. This is in accordance with the suggestion made by the Texas Medical Journal in our July issue, or, rather, it was as near it as was possible, the idea there suggested being to get a "plank" in the platform declaring for a State Board of Health. Dr. M. M. Smith, of Austin, also a member of the Committee on Medical Legislation, attended the convention and pressed the importance of such action on the members of the platform committee. It is earnestly hoped that the governor will give the action of the convention careful attention, and that he will be impressed with the necessity of advising, in his message, that such legislation be enacted as will afford the people of Texas protection from existing sanitary evils and from consequent diseases of local origin, as well as from those of foreign countries, yellow fever, cholera, plague, etc.

